# Development and Validation of an Automated Radiomic CT Signature for Detecting COVID-19

**DOI:** 10.3390/diagnostics11010041

**Published:** 2020-12-30

**Authors:** Julien Guiot, Akshayaa Vaidyanathan, Louis Deprez, Fadila Zerka, Denis Danthine, Anne-Noëlle Frix, Marie Thys, Monique Henket, Gregory Canivet, Stephane Mathieu, Evanthia Eftaxia, Philippe Lambin, Nathan Tsoutzidis, Benjamin Miraglio, Sean Walsh, Michel Moutschen, Renaud Louis, Paul Meunier, Wim Vos, Ralph T. H. Leijenaar, Pierre Lovinfosse

**Affiliations:** 1 Department of Pneumology, University Hospital of Liège, 4020 Liège, Belgium; an.frix@chuliege.be (A.-N.F.); monique.henket@chuliege.be (M.H.); r.louis@chuliege.be (R.L.); 2 Research and Development, Oncoradiomics SA, 4000 Liège, Belgium; akshayaa.vaidyanathan@oncoradiomics.com (A.V.); fadila.zerka@oncoradiomics.com (F.Z.); nathan.tsoutzidis@oncoradiomics.com (N.T.); benjamin.miraglio@oncoradiomics.com (B.M.); sean.walsh@oncoradiomics.com (S.W.); wim.vos@oncoradiomics.com (W.V.); ralph.leijenaar@oncoradiomics.com (R.T.H.L.); 3 The D-Lab, Department of Precision Medicine, Maastricht University, 6229 Maastricht, The Netherlands; philippe.lambin@maastrichtuniversity.nl; 4 Department of Radiology, University Hospital of Liège, 4020 Liège, Belgium; Louis.Deprez@chuliege.be (L.D.); denis.danthine@chuliege.be (D.D.); eeftaxia@chuliege.be (E.E.); Paul.Meunier@chuliege.be (P.M.); 5 Department of Medico-Economic Information, University Hospital of Liège, 4020 Liège, Belgium; mthys@chuliege.be; 6 Department of Computer Applications, University Hospital of Liège, 4020 Liège, Belgium; Gregory.Canivet@chuliege.be (G.C.); smathieu@chuliege.be (S.M.); 7 Department of Infectious Diseases, University Hospital of Liège, 4020 Liège, Belgium; Michel.Moutschen@uliege.be; 8 Department of Nuclear Medicine and Oncological Imaging, University Hospital of Liège, 4020 Liège, Belgium; pierre.lovinfosse@chuliege.be

**Keywords:** artificial intelligence, machine learning, computed tomography, COVID-19, radiomics

## Abstract

The coronavirus disease 2019 (COVID-19) outbreak has reached pandemic status. Drastic measures of social distancing are enforced in society and healthcare systems are being pushed to and beyond their limits. To help in the fight against this threat on human health, a fully automated AI framework was developed to extract radiomics features from volumetric chest computed tomography (CT) exams. The detection model was developed on a dataset of 1381 patients (181 COVID-19 patients plus 1200 non COVID control patients). A second, independent dataset of 197 RT-PCR confirmed COVID-19 patients and 500 control patients was used to assess the performance of the model. Diagnostic performance was assessed by the area under the receiver operating characteristic curve (AUC). The model had an AUC of 0.882 (95% CI: 0.851–0.913) in the independent test dataset (641 patients). The optimal decision threshold, considering the cost of false negatives twice as high as the cost of false positives, resulted in an accuracy of 85.18%, a sensitivity of 69.52%, a specificity of 91.63%, a negative predictive value (NPV) of 94.46% and a positive predictive value (PPV) of 59.44%. Benchmarked against RT-PCR confirmed cases of COVID-19, our AI framework can accurately differentiate COVID-19 from routine clinical conditions in a fully automated fashion. Thus, providing rapid accurate diagnosis in patients suspected of COVID-19 infection, facilitating the timely implementation of isolation procedures and early intervention.

## 1. Introduction

The rapid outbreak of coronavirus disease 2019 (COVID-19), originating from severe acute respiratory syndrome coronavirus 2 (SARS-COV-2) infection, has clearly become a public health emergency of international concern [1]. The outbreak of COVID-19 had a terrible impact on economy and society all around the world. Globally there have been 71,554,018 confirmed cases and 1,613,671 deaths as of 20 December 2020 [2]. The presence of the disease is currently confirmed by reverse-transcription polymerase chain reaction (RT-PCR) [3]. There is, however, evidence that the sensitivity of RT-PCR may not be optimal for the objective of very early detection and early intervention on COVID-19 patients [4]. Due to the limited supply of RT-PCR kits, the lengthy turnaround times, and the emergence of false-negative cases, some experts propose to diagnose suspected cases using the widely available, time-saving and non-invasive imaging approach of chest computed tomography (CT) rather than RT-PCR [5,6]. CT can capture imaging features from the lung, associated with COVID-19 [7], in the early stages of the disease [8]; CT could thus serve as an efficient and effective way to flag, diagnose, and possibly triage COVID-19 patients, in a more timely manner compare to traditional confirmation tests. Despite these advantages, there are several open questions on the use of CT for these purposes [9,10], due to increased radiation exposure of the population and the risk of cross-infection if disinfection is not properly implemented. Notwithstanding these concerns, the use of chest CT for COVID-19 diagnosis needs a proper toolset, to allow clinicians to fully exploit this technology. In the medical imaging domain, artificial intelligence (AI) coupled with machine learning technology has accomplished impressive results due to the intrinsic properties of machine vision [11,12,13,14] and can be leveraged in this scenario. More so, radiomics approach which was already proved to be extremely successful for cancer diagnosis and prognosis [15] might be also applied in this context. Radiomics is the high-throughput mining of quantitative image features from standard-of-care medical imaging that enables data to be extracted and applied within clinical decision support systems to improve diagnostic, prognostic, and/or predictive accuracy [16]. Conceptually, radiomics is a bridge between imaging and precision medicine [17]. In this study, we hypothesize that a radiomics analysis can identify a diagnostic signature for COVID-19 infection, based on standard-of-care chest CT imaging. As a result, we present a fully automated AI framework to detect COVID-19 using chest CT, referred to as COVIA (“coronavirus intelligence artificielle”) and validate its performance in an independent test cohort. This model has been built in a clinical real life environment, the first Belgian wave of COVID-19 infection. This was mainly used for symptomatic patients with the European standard-of-care. Contrary to what is seen in other countries, we used CT scan from all patients reducing the bias found in some studies where clinicians reserved CT only for severe cases.

## 2. Materials and Methods

### 2.1. Ethics

The study has been approved by the local ethics committee of the Centre Hospitalier Universitaire (CHU)-Liège (EC number 116/2020). The institutional review board waived the requirement to obtain written informed consent for this retrospective case series, since all analyses were performed on de-identified (i.e., pseudo-anonymized) data and there was no potential risk to patients.

### 2.2. Subjects

Three cohorts of patients were included retrospectively in this study. Cohorts came from two sites (CHU Sart-Tilman and CHU Notre Dame des Bruyères) in Liège, Belgium. The first cohort (label: COVID) consists of all patients with COVID-19 infection confirmed by RT-PCR that underwent chest CT imaging before 28 March 2020. The second cohort (label: Control) consisted of consecutive patients that underwent chest CT imaging between 1 October 2019 and 24 October 2019, which ensures that none of these patients were infected by COVID-19. The third cohort (label: Test) consisted of 697 consecutive patients that underwent chest CT imaging between 12 August 2019 and 6 April 2020. The Test cohort presents no overlap with COVID and Control cohorts and was acquired at a different time point. Within this cohort, 197 patients had RT-PCR confirmed COVID-19, whereas the remaining 500 patients tested negative for COVID-19. The first (COVID) and second (Control) cohort were used for model development, the third cohort (Test) was used as an independent test set. No other inclusion or exclusion criteria were considered while collecting the data. This resulted in sets of CT images from either COVID-19 infected patients or non-infected patients (normal and with a variety of diseases) representing real life conditions.

### 2.3. Radiomics

**Figure 1:**
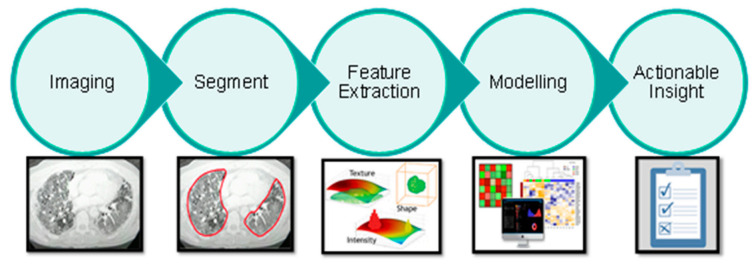
Schematic representation of the radiomics analysis steps: **Imaging**: chest CT scans of healthy and COVID-19 infected patients were collected and divided between training and testing cohort. **Segment**: the scans were automatically segmented to delineate the region of interest in the lung. **Feature extraction**: hand-crafted radiomics feature were extracted from the region of interest. **Modelling**: the radiomics features were used to train the AI model and the performances were validated in the test set. **Actionable insight**: the model discrimination performances were assessed in term of accuracy, sensitivity, specificity, negative predictive value (NPV) and positive predictive value (PPV).

Radiomics focuses on improvements of image analysis, using automated high-throughput extraction of large amounts (200+) of quantitative features from medical images. The hypothesis is that quantitative analysis of medical image data via automatic or semi-automatic software can provide more and better information than that of a physician [18,19]. The schematic representation in Figure 1 depicts the radiomics workflow applied in this study. The following sections will detail each step in the workflow.

### 2.4. Imaging

All CT images used in the study were acquired on one of five multidetector CT scanners (Siemens Edge Plus (2), GE Revolution CT (1), GE Brightspeed (2)) available at the sites. Since CT images were collected retrospectively, no standardized scan protocol was available over the complete dataset. In order to prevent excess variability in the imaging used for model generation, the following criteria for radiomic analysis were used:

- Lungs completely visible in the scan;
- Slice increment less than 1.5 mm;
- No missing slices;
- For GE scans: STANDARD reconstruction kernel;
- For Siemens scans: B30-range reconstruction intervals;

### 2.5. Lung Segmentation

**Figure 2:**
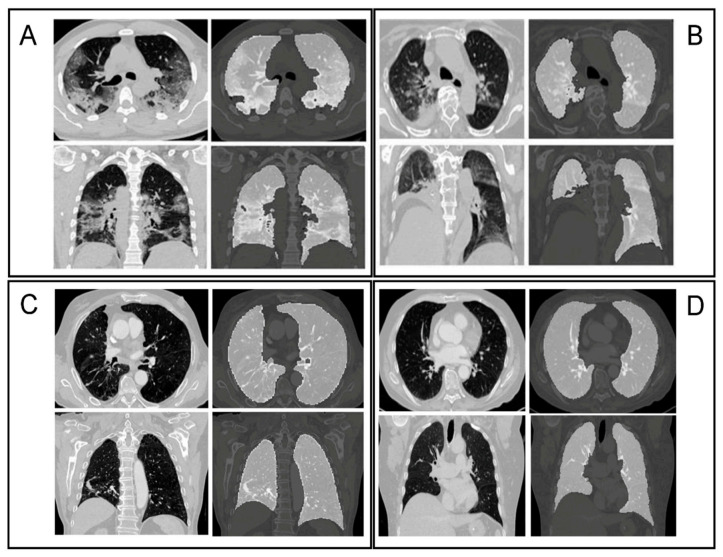
Axial and coronal slices with accompanying segmentation masks. (**A**) Typical aspect of COVID-19 pneumonia characterized by bilateral multilobe ground-glass opacities of peripheral/subpleural distribution, with intralesional reticulations, presenting a “crazy paving” aspect. Subpleural atelectasis and retraction bronchiectasis, typical of organizing pneumonia can also be found; (**B**) a typical aspect of COVID-19 pneumonia, with posterior right lower lobe condensation and retraction of the ipsilateral diaphragm. Central and peripherical ground-glass opacities in right lower lobe, right upper lobe and left upper lobe; (**C**) typical chronic obstructive pulmonary disease (COPD) chest computed tomography (CT) characterized by severe centrilobular and para-septal emphysema, associated with cylindrical bronchiectasis and bronchial walls thickening. Right peripherical upper lobe tree in bud pattern seen in bronchiolitis. Middle lobe crescent-shaped atelectasis condensation; (**D**) normal chest CT.

The lungs were segmented as a single structure using RadiomiX (OncoRadiomics SA, Liège, Belgium) based on Convolutional Neural Networks (CNN) by combining 2D and 3D architectures. The predicted segmentations of each architecture are assembled and the intersection constitutes the final lung segmentation which is used for extraction of radiomics features. Figure 2 shows example segmentations for four patients from both the COVID and Control groups. Complete details on the segmentation methods can be found in SI (see Appendix S1—Segmentation protocols).

### 2.6. Feature Extraction

For each patient, 166 image features were extracted from the lung segmentation using RadiomiX (OncoRadiomics SA, Liège, Belgium) based upon quantitative image analysis technology. The extracted features comprised first order and intensity histogram statistics, texture (gray-level co-occurrence, gray-level run-length, gray-level size-zone, gray-level distance-zone, neighbourhood gray-tone difference and neighbouring gray-level dependence matrix based features), and shape. A bin width of 25 Hounsfield units was used for image intensity discretization. No further image pre-processing was performed. The mathematical descriptions of all features are reported in [17].

### 2.7. Modelling

For model development, multivariable logistic regression with Elastic Net regularization was performed in the training data set. Highly correlated features, features with near zero variance and linear combinations between features were first eliminated from further analysis. For each highly correlated feature pair (Pearson correlation coefficient ρ > 0.9), the variable with the largest mean absolute correlation with all remaining features was removed. Model training was performed using 100 times repeated 10-fold cross-validation to select the optimal model hyperparameters, optimizing for area under the receiver operating characteristic curve (AUC). All features were standardized before modelling. To further reduce the chance of overfitting to the training data, we selected the simplest candidate model (i.e., the model with the fewest non-zero coefficients) within one standard error of the best performing model. Model performance was validated in the test data set. Here, the AUC was used to assess model performance in discriminating between COVID-19 positive and COVID-19 negative patients. Additionally, a hard classification was performed (i.e., classifying patients as either COVID-19 positive or negative) by applying different decision thresholds on the continuous scores (probabilities) predicted by the model on the test data set. Classification performance was then assessed by determining accuracy, sensitivity, specificity, negative predictive value (NPV) and positive predictive value (PPV) for each decision threshold, assuming a disease prevalence of 15%. All statistical analysis was performed in R (R Core Team, Vienna, Austria version 3.6.2).

## 3. Results

### 3.1. Study Population

Table 1 lists the study population characteristics for the model development data (the COVID and Control cohorts), and the independent test dataset (the Test cohort), as well as the main CT findings as scored by radiologists. For the model development data, the COVID-19 positive and control patients have a similar mean age and male/female distribution. For the COVID-19 infected patients 69% needed O_2_ at admission, resulting in 37% of patients ending up in the ICU. A total of 17% of COVID-19 patients needed mechanical ventilation and 4% died. The comorbidity summary for the COVID-19 patients is presented in Table 2. For the independent test data set, the COVID-19 positive and control patients have a similar mean age and male/female distribution and 41% of the COVID-19 patients were admitted to the ICU.

### 3.2. Data Curation

**Figure 3:**
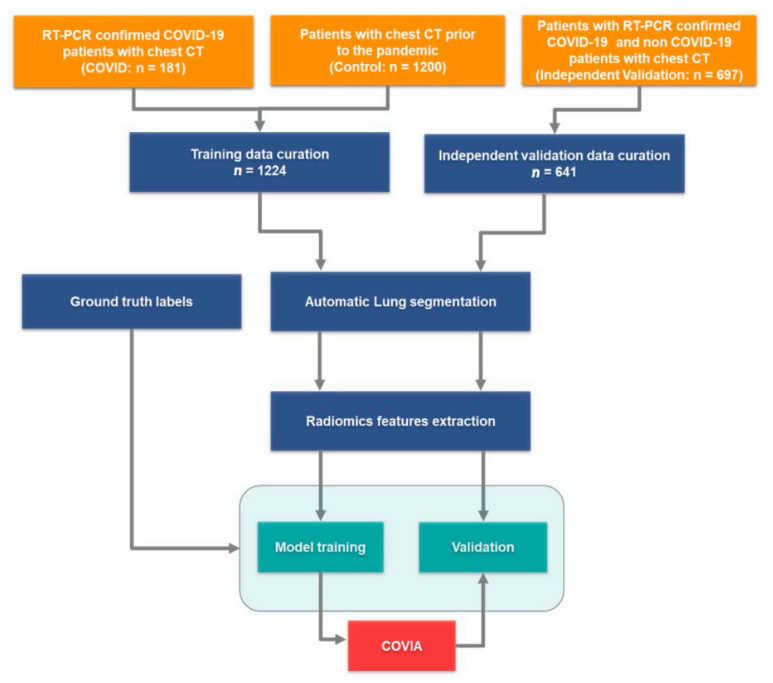
Flow diagram: Training and validation data were collected, the COVID and Control cohorts were combined. Lungs were segmented from both the training and validations datasets, respectively, and radiomics features were extracted. The independent validation data was used to test the performance of coronavirus intelligence artificielle (COVIA) with unseen patient CTs.

After an automated quality check on the inclusion criteria, CT images and lung segmentations for a total number of 1224 patients for model development and 641 patients for independent model testing were included for further processing. A flow chart describing the overall workflow from data collection to model training and testing is shown in Figure 3.

### 3.3. COVID-19 Infection Status Prediction

**Figure 4:**
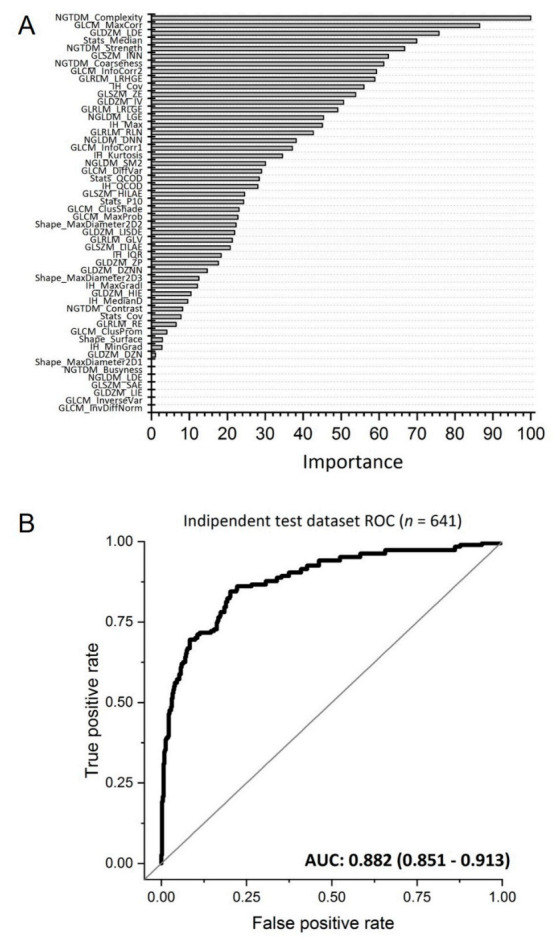
(**A**) Features with a non-zero regression coefficient in the model and their importance, based on their absolute regression coefficient, and scaled between 0 and 100; (**B**) ROC plot illustrating the performance (black curve) of the AI framework to discriminate between COVID-19 positive and negative cases in the independent test data set with an area under the receiver operating characteristic curve (AUC) of 0.882 (95% CI: 0.851–0.913).

**Figure 5:**
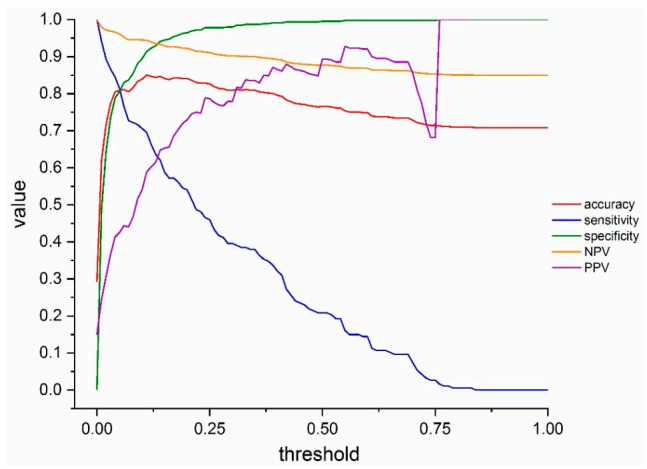
Classification performance plot. The classification performance in the test dataset, assuming a disease prevalence of 15%, in terms of accuracy (red line), sensitivity (blue line), specificity (green line), NPV (orange line) and PPV (purple line) for different decision thresholds.

**Figure 6:**
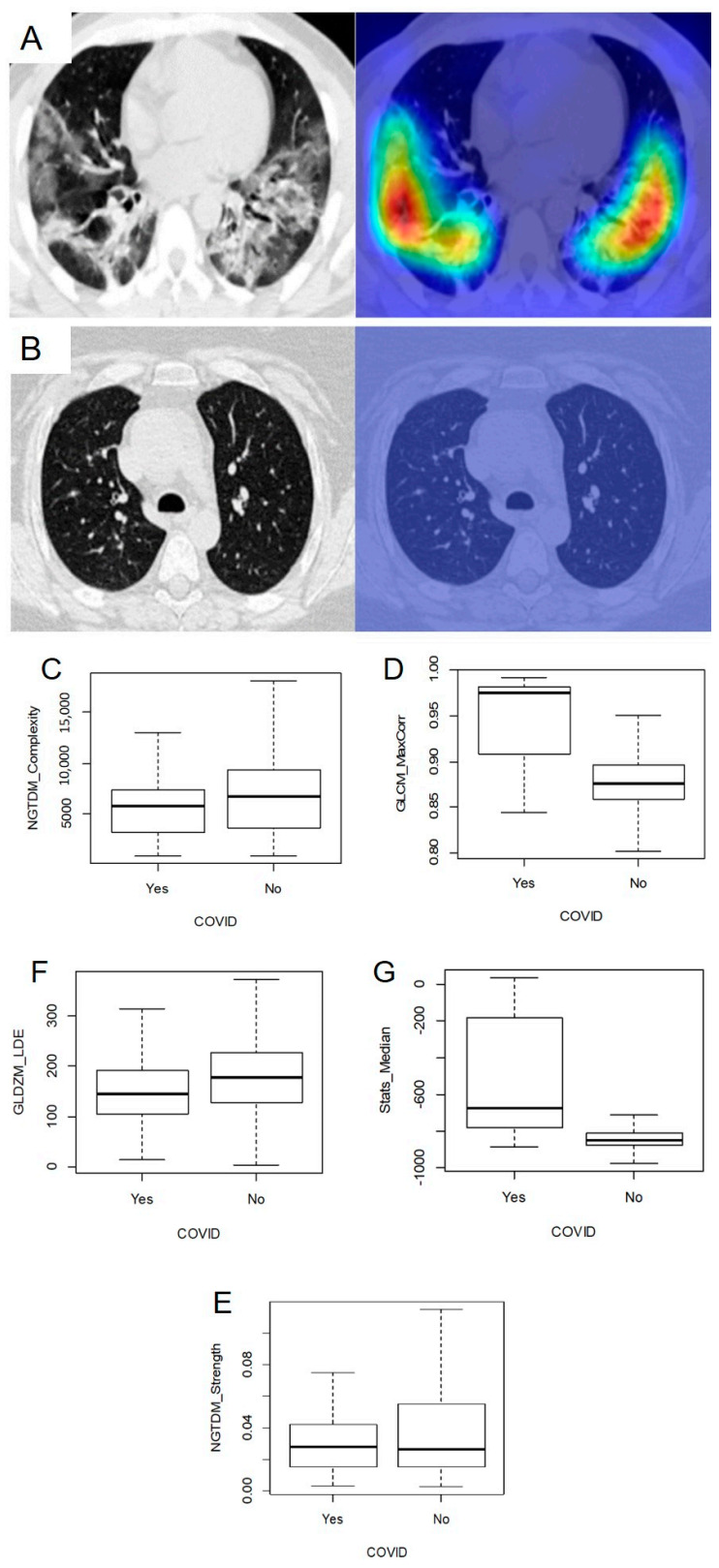
Chest CTs of a typical COVID-19 positive patient (**A**): original scan—left; heat-map—right) with evident reticulation, ground glass opacities and condensations compared to a healthy patient CT scan (**B**): original scan—left; heat-map—right). Heat-maps underline the more relevant areas for model prediction. Box plots comparing the distribution of the top 5 features among COVID and non-COVID cases ((**C**)—NGTDM_Complexity; (**D**)—GLCM_MaxCorr; (**E**)—NGTDM_Strenght; (**F**)—GLDZM_LDE; (**G**)—Stats_Median).

The final prediction model included 45 radiomics features with a non-zero regression coefficient. Included features and their importance, in terms of the absolute regression coefficient, are shown in Figure 4A while the Receiver operating characteristic curve ROC curve for the independent test data set is shown in Figure 4B. The corresponding AUC value for discriminating between COVID-19 positive and negative cases is 0.882 (95% CI: 0.851–0.913). Assuming a disease prevalence of 15%, the classification performance in the test dataset, in terms of accuracy, sensitivity, specificity, NPV and PPV for different decision thresholds are shown in Figure 5. For example, a threshold of 0.11 corresponds to the optimal decision threshold in terms of the Youden Index, when considering the cost of false negatives twice as high as the cost of false positives. This particular decision threshold results in an accuracy of 85.18%, a sensitivity of 69.52, a specificity of 91.63%, a NPV of 94.46% and a PPV of 59.44% for COVID-19 classification. Figure 6 depicts a chest CT of a typical COVID-19 positive patient (Figure 6A), and a normal chest CT (Figure 6B) alongside their corresponding heat-maps extracted from an end-to-end conventional black-box AI-based model trained to screen COVID. The heatmaps were obtained from a conventional CNN model based on VGG16 architecture trained to classify COVID from other CT images. A technique called Gradient based localization [20] was used to obtain the heatmaps which explain the model’s decision to classify the image in Figure 6A as a COVID case.

Table 3 lists the values of the top five radiomics features and model scores (SCORE) of cases depicted in Figure 6A,B. The top five features are: a measure of texture complexity, quantifying non-uniformity and sudden changes in intensity values within the region of interest (NGTDM_Complexity; Neighborhood gray tone difference matrix, Complexity); a texture measure of correlation of the grey-level co-occurrence matrix (GLCM_MaxCorr; grey level co-occurrence matrix, maximal correlation coefficient); a texture measure emphasizing larger distances to the edge of the region of interest of connected voxels of similar intensity values (GLDZM_LDE; grey level distance zone matrix, Large distance emphasis); the median image intensity in the lungs (Stats_Median; First order statistics, Median); a measure of texture strength, quantifying how definable or visible the texture is within the image (NGTDM_Strength; Neighborhood gray tone difference matrix, Strength). Figure 6C–G report the box plots for the distribution of features among the COVID and non-COVID groups.

## 4. Discussion

COVID-19 has spread rapidly across the globe and the rate of infection is accelerating. Therefore, rapid and early diagnosis of the disease is essential for intervention and swift isolation of patients in order to prevent the spread of the virus. RT-PCR is considered the “gold standard” for COVID-19 identification However, there are reports of false-negatives occurring which are eventually confirmed as true-positive by repeated swab tests [21]. False negatives can be a significant problem in high-throughput settings operating under severe pressure [22]. The correct operation of the test is crucial and there is ambiguity with respect to the kinetics of SARS-CoV-2 viral shedding, thus the timing of the test may very well dictate the result. Furthermore, it is also unclear what kind of clinical sample is most appropriate as nasopharyngeal swabs may offer greater consistency than sputum samples [23]. When considering the limited supply of RT-PCR kits, the growing backlog and the likely increasing pressure and turnaround times in laboratories along with the issues pertaining to false-negatives, prompted the experts to suggest that to diagnose suspected cases using the widely available, time-saving and non-invasive imaging approach of chest CT is justified. This approach has been proved useful in sensitively and specifically identify COVID-19 patients [24]. We have shown that our model is able to achieve a high NPV (94.46%), which provides further justification for using CT imaging-based diagnosis as primary tool for COVID-19 patient management.

Whereas similar studies in COVID-19 focus mainly on the detection of various diseased regions (including ground-glass opacification, consolidation, bilateral involvement, peripheral and diffuse distribution amongst others) in the lung [25,26,27], our approach performs an easy segmentation of the lungs as one single structure, which is by far an easier task to automate with AI. Features for quantitative image analysis are extracted from this whole lung structure and subsequently used for prediction model application and COVID-19 infection status classification. In the end this constitutes a fully automated clinical decision support tool for the diagnosis of COVID-19, which is able to provide an objective, robust (i.e., no user variability) and easy to interpret classification (yes–no) of COVID-19 infection status. The complete workflow takes between 40–60 s, providing a rapid and accurate diagnosis in patients with suspicion of COVID-19 infection, facilitating the timely implementation of isolation procedures and early intervention.

We developed a machine learning model that is able to discriminate between COVID-19 positive and negative patients, and which has been trained and validated using a regularized logistic regression model. Elastic net logistic regression has been used, for its relatively straightforward interpretation of linear models and its demonstrated discriminative performance [26]. The continuous prediction scores of the model can be utilized for binary classification of patients (COVID-19 infected or not). Given this continuous output of the underlying model, it is possible to optimize the decision threshold used for hard classification based on more appropriate prevalence and costs of misclassification, which may vary, for instance, per geographic area. Although this study focuses solely on using image data for COVID-19 diagnosis, it is possible to imagine that, combining the model’s continuous score with other clinical data, an even more accurate determination of overall probability of diagnosis could be achieved.

We plan to test the capability of the AI algorithm in the diagnosing of COVID-19 against that of radiologists in a virtual clinical trial setting. This aspect is vital in the context of incidental findings, which are of increasing relevance [27]. An automated AI solution could be helpful in assisting the accurate identification of potentially COVID-19 positive patients, alerting the radiologist who must prioritize the reading of this examination and the radiology department that a “clean machine” now requires decontamination.

A general objection of AI methods is the lack of transparency and interpretability. This is not the case with our approach, as “handcrafted” radiomics features are explicitly defined and linked to clearly specified regions of interest within the images, driving the decision of the algorithm. Thus, clearly and intelligibly quantifying the image phenotype, which has also been shown to provide a means of connecting to the underlying biology [28]. The interpretability of an AI based classifier’s decision is limited to highlighting image regions contributing to the decision, which allows only for qualitative interpretation (i.e., human/expert interpretation of these image regions). Our model proves to be more interpretable and explainable as the (top) features are associated to clearly pre-defined regions of interest and their values can be directly compared between different patients, as well as further interpreted based on their unambiguous mathematical definitions. For instance, the features listed in Table 3 clearly show difference in values between normal and COVID patients CTs. Hence, those features quantify a radiomics phenotype linked to the bilateral multilobe ground-glass opacities of peripheral/subpleural distribution, with intralesional reticulations seen on this typical COVID-19 positive chest CT.

Given the rapid development of serum-based tests for COVID-19, a critical contextualization is important. Serum analysis is dependent on logistics and takes a relatively long time to deliver results when compared with AI (near instantaneous). In the best case scenario serum takes hours, in the worst case several days [22]. Furthermore, serum analysis is practically limited to large centres with advanced biotechnology capabilities in developed countries (small centres have increased logistical challenges). In the case of an emergency procedure (e.g., surgery), the probable COVID-19 status of the patient must be immediately addressed in order to safeguard the hospital with respect to transmission. Considering beyond the current pandemic phase that we are in, serum analysis offers little value in the way of incidental findings as clinicians will be less pro-active in ordering tests to determine COVID-19 infection. With respect to RT-PCR detection [29], the positive rate of the 2019-nCoV nucleic acid test of a nasopharyngeal swab is 38% (180/472 times), the positive rate of the 2019-nCoV nucleic acid test of the sputum is 49% (148/304 times), the positive rate of the blood 2019-nCoV nucleic acid test is 3% (4/132 times), and the positive rate of the 2019-nCoV nucleic acid test of faeces is 10% (24/244 times). The positive rate of the 2019-nCoV nucleic acid detection in anal swabs is 10% (12/120 times). A meta-analysis [30] showed the pooled sensitivity was 94% (95% CI: 91%, 96%) for chest CT and 89% (95% CI: 81%, 94%) for RT-PCR. The pooled specificity was 37% (95% CI: 26%, 50%) for chest CT. The prevalence of COVID-19 outside China ranged from 1% to 23%. The PPV ranged from 2% to 31%, and the NPV ranged from 95% to 100%. COVIA was tested against an assumed prevalence of 15% and the classification results indicate competitive performance.

In the last few months the literature about AI assisted diagnosis and classification of COVID-19 infection has boomed like never before [31,32]. Many relevant papers have been published, reporting multicentric validation studies with remarkable performance [33,34,35], along with new insights into the clinical aspect of CT scan COVID-19 characteristics [36]. In this fast-evolving field, where much innovation sometimes goes along with overly enthusiastic reports [37], our method has several advantages over other reported AI based diagnostic tools: first of all, the automatic segmentation of the whole lung does not require human input, speeding up the process and unburdening medical staff. More important, however, is the use of robust and validated radiomics features, compared to other parameters used in other approaches like consolidation and ground-glass opacity alone [38,39,40], which are not specific for the disease [9].

Compared to other radiomics signatures published in the last months [41,42], our signature was trained and tested on a wider dataset, acquired at different time points, to account for the small variability that might be present in scan acquisition at different dates. This is considered a more reliable strategy [43] as it closely mimics what happens in a real world clinical scenario. The robust testing strategy of the model, coupled with the interpretability of the radiomics features, assures the reliability of the proposed model.

It is worth pointing out, however, that the study has still some limitations. Firstly, COVID-19 is caused by SARS-CoV-2 and may have similar imaging characteristics as pneumonia caused by other types of viruses. However, due to the lack of laboratory confirmation of the aetiology for each of these cases, we were not able to select other viral pneumonias for comparison in this study. Although our Control group of non-COVID-19 patients contains several patients (see CAP in Table 1, 12.5%) with pneumonia (either viral, bacterial or pneumonia from any other cause), it would be desirable to test the performance of our algorithm in distinguishing COVID-19 from other viral pneumonias that have RT-PCR confirmation methods for the viral agent.

Moreover, the population of patients with COVID-19 was selected after clinical evaluation of patients with respiratory symptoms such as dyspnea and desaturation. The degree of severity justified the fact that imaging analysis was left to clinical judgement and depending on local resources [23]. Therefore, COVIA was partially developed in a population of patients with disease at the moderate to severe end of the spectrum. Further analysis into the benefit, if any, of COVIA for patients with mild or no symptoms is required.

Future work is planned to collect additional chest CTs to externally validate the performance of our algorithm in an international multi-centre prospective external validation to produce evidence level 1 [30] for the clinical utility of COVIA. The study protocol is in development and will be registered on clinicaltrials.gov.

Ultimately, this study was focused on diagnosis whereas prognosis on the future disease trajectory is an even more urgent unmet clinical need that would enable improved resource management (including management decisions regarding the allocation of resources). This is the next step for our collaborative research.

## 5. Conclusions

Benchmarked against RT-PCR confirmed cases of COVID-19, our AI framework can accurately detect COVID-19. Thus, it provides rapid accurate diagnosis in patients with suspected of COVID-19 infection, facilitating the timely implementation of isolation procedures and early intervention. The proposed model, trained on a diverse and robust dataset, showed good performance (AUC of 0.882) with the added valuable of being explainable, linking the radiomics results with real clinical evidence, like lung abnormalities (ground glass opacities, consolidations and others). This approach will be extended and improved, including the distinction between different types of pneumonia, streamlining the staging and therapy planning of patients. A further improvement could comprise the creation of a prognostic model along with the diagnostic one, to assess severity of newly admitted patients and the probability of developing serious symptoms or admission to the ICU.

## Figures and Tables

**Table 1:** Summary of patient characteristics (age, gender and CT findings scored by radiologist) per cohort.

	Training Set (*n* = 1381)		Independent Validation Set (*n* = 697)	
	CONTROL (*n* = 1200)	COVID (*n* = 181)	CONTROL (*n* = 500)	COVID (*n* = 197)
Age (years)	63.8 ± 14.4	64.4 ± 15.8	64.2 ± 14.0	69.1 ± 13.3
Gender (% Male)	52	56	51	56
Normal (%)	33	4.41	25.2	25
Neoplasia (%)	8.73	0	0	0
CAP (%)	12.50	8.10	6.6	8.6
COPD (%)	26	19.33	33.4	11.7
Isolated pleurisy (%)	6.2	1.10	4.2	4
Pulmonary embolism (%)	0.77	1.10	0	0
Nodule (%)	19	6.62	17.2	6.6
Chronic inflammation (%)	8.48	5.52	13.6	3
Pneumothorax (%)	0.68	0	0.6	0
Isolated atelectasis (%)	3.68	3.31	5.4	1.0

**Table 2:** Baseline characteristics of the COVID-19 patients used for model training.

Any Comorbidity	COVID Training Set (*n* = 181)
Neoplasia (%)	23.7
Acute Respiratory Failure (%)	26.7
Heart disorder (%)	15.9
Hypertension (%)	6.8
Diabetes (%)	4.7
Chronic renal failure (%)	1.8
Obesity (%)	0

**Table 3:** Top 5 radiomics features and model scores of cases depicted in Figure 6.

	Normal Chest CT	COVID-19 Positive
NGTDM_Complexity	7794.055	1147.344
GLCM_MaxCorr	0.8684842	0.9147317
GLDZM_LDE	143.07153	57.53219
Stats_Median	−839	−755
NGTDM_Strength	0.033166649	0.008062981
SCORE	0.01119137	0.765581

## Data Availability

Patient consent was waived since all analyses were performed on de-identified (i.e., pseudo-anonymized) data and there was no potential risk to patients. The study has been approved by the local ethics committee of the CHU-Liège (EC number 116/2020). The study was conducted according to the guidelines of the Declaration of Helsinki, and approved by the Institutional Review Board (or Ethics Committee) of CHU-Liège (EC number 116/2020).

## Supplementary Materials

The following are available online at https://www.mdpi.com/2075-4418/11/1/41/s1, Appendix S1—Segmentation protocols

## Abbreviations

PPV	Positive Predictive Value
NPV	Negative Predictive Value
AUC	area under the receiver operating characteristic curve
RT-PCR	Reverse transcription polymerase chain reaction
SARS-COV-2	severe acute respiratory syndrome coronavirus 2
AI	Artificial intelligence
COVIA	coronavirus intelligence artificielle
CT	computed tomography
