# Supplementary material for: Development and Validation of an Automated Radiomic CT Signature for Detecting COVID-19
Source: Diagnostics (Basel). 2020 Dec 30;11(1):41. doi: 10.3390/diagnostics11010041 (PMC7823620; doi:10.3390/diagnostics11010041)
Supplement: Supplementary file 1 [file diagnostics-11-00041-s001.pdf]

Supporting Information

Appendix S1 – Segmentation protocols

1. The lungs were segmented as a single structure using RadiomiX (Oncoradiomics SA, Liège, Belgium) based on convolutional neural networks by combining 3D and 2D architectures. Details on both these architectures are given below.

1.1 3D lung segmentation

This model architecture consists of a 3D U-Net [1] with residual blocks [2] in the encoder part of the network. Publicly available data from the cancer imaging archive [3] was used to train and validate the model. The specific dataset [4] contains CT scans of 422 confirmed non-small cell lung cancer cases, along with manual segmentations of the left and right lungs. The segmentations were performed by an experienced radiologist and these segmentations were used as a reference standard. The data was randomly partitioned into a training set (n = 322), a tuning set (n = 50), and a test set (n = 50). In order to generate homogeneous CT volumes as input for the model, the following pre-processing steps were performed. All the volumes were resized to 160 x 160 x 448 along the x, y and z axis .and image intensities were clipped at a window width of 1500 HU and a window level of -600 HU (i.e., a standard lung CT window level settings).

The following data augmentations were performed to avoid overfitting [5] on the training dataset:

1. Flipping in different directions: up and down, left and right
2. Randomly resampling volumes to varying voxel sizes and resize to dimensions (160 x 160 x 448)
3. Rotating (10-30 degrees) onto the left or right direction

4. Reversing the sequence of axial slices

The model was trained with the pre-processed volumes and their corresponding reference labels, using Jaccard loss [6] as an objective function. Here, the loss is calculated in a mini batch of two images per iteration. The network was trained for 10 epochs and at the end of each epoch the Jaccard loss was calculated on the model's predictions to ensure validation loss convergence.

1.2 2D lung segmentation

The 2D lung segmentation model architecture is based on a 2D Feature Pyramid Network [7] adapted with ResNext blocks [8] in the encoder. The model was trained and validated on the following datasets,

1. Publicly available dataset with 888 CT scans and the corresponding reference annotations for lungs available from LUNA16 challenge [9]
2. Publicly available data from the cancer imaging archive [34] containing CT scans of 422 confirmed non-small cell lung cancer cases, along with manual segmentations of the left and right lungs. The segmentations were performed by an experienced radiologist and these segmentations were used as a reference standard.

The network was trained with the 2D axial slices clipped at a window width of 1500 HU and a window level of –600 HU and with their corresponding reference labels. The network's weights were updated by using the Adam optimizer at an initial learning rate of 1e-5 [10]. The model was trained using customized Jaccard loss [6] as an objective function where the loss is calculated in a mini batch of 8 images per iteration. The

network was trained for 5 epochs and at the end of each epoch, the Jaccard loss was calculated on the model's predictions to ensure validation loss convergence.

The predicted segmentations of each architecture (i.e., the segmentation output from both the 3D and the 2D segmentation models) were ensembled and the intersection constitutes the final total lung segmentation which is used for extraction of radiomics features. The deep learning-based lung segmentation achieved a mean Dice similarity coefficient score of 0.92 across the publicly available datasets which indicates adequate precision (i.e. no significant over or under segmentation).

References

1. Çiçek Ö, Abdulkadir A, Lienkamp SS, Brox T, Ronneberger O. 3D U-net: Learning dense volumetric segmentation from sparse annotation. In: *Lecture Notes in Computer Science (Including Subseries Lecture Notes in Artificial Intelligence and Lecture Notes in Bioinformatics)*. ; 2016. doi:10.1007/978-3-319-46723-8_49

2. He K, Zhang X, Ren S, Sun J. Deep residual learning for image recognition. In: *Proceedings of the IEEE Computer Society Conference on Computer Vision and Pattern Recognition*. Vol 2016-December. IEEE Computer Society; 2016:770-778. doi:10.1109/CVPR.2016.90

3. Prior F, Smith K, Sharma A, et al. The public cancer radiology imaging collections of The Cancer Imaging Archive. *Sci data*. 2017;4:170124. doi:10.1038/sdata.2017.124

4. NSCLC-Radiomics - The Cancer Imaging Archive (TCIA) Public Access -

Cancer Imaging Archive Wiki.

5. Caruana R, Lawrence S, Giles L. Overfitting in neural nets: Backpropagation, conjugate gradient, and early stopping. In: *Advances in Neural Information Processing Systems*. ; 2001.

6. Bertels J, Eelbode T, Berman M, et al. Optimizing the Dice Score and Jaccard Index for Medical Image Segmentation: Theory & Practice. *Lect Notes Comput Sci (including Subser Lect Notes Artif Intell Lect Notes Bioinformatics)*. 2019;11765 LNCS:92-100. doi:10.1007/978-3-030-32245-8_11

7. Lin TY, Dollár P, Girshick R, He K, Hariharan B, Belongie S. Feature pyramid networks for object detection. In: *Proceedings - 30th IEEE Conference on Computer Vision and Pattern Recognition, CVPR 2017*. Vol 2017-January. Institute of Electrical and Electronics Engineers Inc.; 2017:936-944. doi:10.1109/CVPR.2017.106

8. Xie S, Girshick R, Dollár P, Tu Z, He K. Aggregated residual transformations for deep neural networks. In: *Proceedings - 30th IEEE Conference on Computer Vision and Pattern Recognition, CVPR 2017*. Vol 2017-January. Institute of Electrical and Electronics Engineers Inc.; 2017:5987-5995. doi:10.1109/CVPR.2017.634

9. Setio AAA, Traverso A, de Bel T, et al. Validation, comparison, and combination of algorithms for automatic detection of pulmonary nodules in computed tomography images: the LUNA16 challenge. *Med Image Anal*. 2016;42:1-13. doi:10.1016/j.media.2017.06.015

10. Kingma DP, Ba JL. Adam: A method for stochastic optimization. In: *3rd*

International Conference on Learning Representations, ICLR 2015 - Conference Track Proceedings. ; 2015.
